# Antibiotic Resistance and Genomic Diversity of Methicillin-Resistant *Staphylococcus aureus* Clonal Complex 45 Isolates in Kuwait Hospitals

**DOI:** 10.3390/antibiotics15040362

**Published:** 2026-04-01

**Authors:** Samar S. Boswihi, Tina Verghese, Edet E. Udo

**Affiliations:** Department of Microbiology, College of Medicine, Kuwait University, P.O. Box 24923, Safat 13110, Kuwait; samar.boswihi@ku.edu.kw (S.S.B.); tina.verghese@ku.edu.kw (T.V.)

**Keywords:** MRSA, genotyping, antibiotic resistance, clonal complex 45

## Abstract

**Background/Objectives:** Methicillin-resistant *Staphylococcus aureus* (MRSA) causes hospital- and community-acquired infections. MRSA is a highly diverse strain that includes several epidemic clones, including CC45. A previous study conducted among MRSA isolates in Kuwait identified CC45 in two isolates in the early 2000s. This study provides an update on the prevalence and molecular characteristics of CC45 among MRSA isolates in Kuwait hospitals, during 2016–2022. **Methods:** A total of 13,276 MRSA isolates were collected during 2016–2022 and typed using antibiogram, DNA microarray, Staphylococcal protein A (*spa*) typing, pulsed-field gel electrophoresis (PFGE), and multi-locus sequence typing (MLST). **Results:** CC45 was detected in 87 (0.65%) of the 13,276 MRSA isolates. The isolates were resistant to fusidic acid (n = 71), erythromycin (n = 16), and inducible clindamycin resistance (n = 15). Twenty-one isolates were resistant to multiple antibiotics. *Spa* typing identified 19 types, with t362 (n = 35) and t132 (n = 27) as the dominant types. DNA microarray identified seven genotypes with CC45-MRSA-[IV + fus] (n = 36) and CC45-MRSA-[VI + fus] (n = 30) as the dominant types. MLST identified six sequence types (STs): ST7119, ST508, ST45, ST46, ST9548, and ST10699. PFGE clustered the isolates into two major types, A and B, with type A being the major type (n = 83), mostly consisting of CC45-MRSA-[IV + fus] isolates. The CC45-MRSA-[IV + fus] and CC45-MRSA-[VI + fus] genotypes were detected throughout the study period (2016–2022), whereas the other genotypes were detected less frequently. **Conclusions:** The CC45-MRSA circulating in Kuwait hospitals comprises genetically diverse isolates that may have originated from different sources. The emergence of multidrug resistance among the isolates poses challenges for therapy and infection prevention.

## 1. Introduction

Methicillin-resistant *Staphylococcus aureus* (MRSA) is a major pathogen that causes community-acquired and hospital-acquired infections worldwide [1]. According to the World Health Organization (WHO), MRSA continues to be a high-priority pathogen since it continues to cause infections that result in a high rate of mortality and morbidity among humans [2]. The rapid development of antimicrobial resistance, whether through genetic acquisition or mutations, further compounds the challenges of treating and managing MRSA infections [3].

Molecular characterization of MRSA from various geographic locations, using different epidemiological typing methods, has revealed diverse lineages grouped into clonal complexes and sequence types. These studies have identified clones capable of spreading widely across several countries, as well as those with limited geographic spread [1]. MRSA isolates belonging to clonal complexes (CCs) CC1, CC5, CC8, CC22, CC30, CC45, CC80, and CC97 have a wide geographic distribution, whereas isolates belonging to CC93 and CC59 have demonstrated restricted geographic distribution across several countries [1,4,5].

Clonal complex (CC) 45 is a cluster of *S. aureus* isolates defined by multilocus sequence typing (MLST) sequence type (ST) 45 and closely related sequence types (STs). CC45 comprises methicillin-susceptible *S. aureus* (MSSA) and methicillin-resistant *S. aureus* (MRSA), including community-acquired (CA) and hospital-acquired (HA) strains, as well as clinical and commensal strains [4,5]. Some CC45 MRSA clones are associated with invasive infections, including bloodstream infections and endocarditis [5,6,7].

MRSA belonging to CC45 has a global spread. It has been widely reported in Australia [4,8,9], Europe [10,11,12,13], North America [5,6,14], South America [15], and Asia [7,16,17,18,19]. A CC45 lineage designated ST45-MRSA-II, or MRSA-USA600, is widely distributed in North America and countries outside North America, including Hong Kong and Australia [5]. USA600 is a significant cause of endocarditis and bloodstream infections with a high mortality rate in North America [6,20]. Another CC45-MRSA lineage, ST45-MRSA-IV, also known as Berlin-IV or the Berlin epidemic strain, was initially isolated in Berlin hospitals in 1993 and later became a dominant clone across large areas of Germany [21]. Furthermore, ST45-MRSA-IV and ST45-MRSA-V, initially reported among the aboriginal communities in Western Australia, were also found in other Australian regions [22].

In addition to its association with human infections, ST45 has been isolated from animals [23,24] and livestock [25]. As the transmission of potential pathogens between animals and humans, either via direct contact or the food chain, is a rising public health threat, the presence and diversity of ST45 should be carefully monitored [5].

Although the CC45-MRSA clone is widely distributed in Europe, North America, Australia, and, more recently, Asia, it is less commonly reported in the Gulf Cooperation Council (GCC) countries [26]. In Kuwait, sporadic CC45-MRSA isolates were reported in MRSA collections from 2001 to 2005 [27]. Since then, no information has been available on their prevalence and molecular characteristics in Kuwait. This study aimed to provide an update on the prevalence and molecular characteristics of CC45-MRSA, the carriage of genes encoding virulence factors, and antibiotic resistance from 2016 to 2022, and to monitor changes in its distribution over the study period.

## 2. Results

### 2.1. Identification of CC45-MRSA Isolates

DNA microarray analysis of 13,276 MRSA isolates collected from 2016 to 2022 identified 87 isolates belonging to CC45. This study focused on the phenotypic and genotypic characterization of the CC45-MRSA isolates. The CC45 isolates were cultured from nasal swabs (n = 37), blood (n = 6), groin (n = 6), wound swabs (n = 12), high vaginal swabs (n = 4), tissue (n = 2), tracheal aspirates (n = 5), throat swabs (n = 3), ear swabs (n = 3), sputum (n = 2), semen (n = 1), fluid (n = 1), and unspecified samples (n = 5).

### 2.2. Antibiotic Resistance Phenotype and Genotype in CC45-MRSA Isolates

All 87 CC45-MRSA isolates were resistant to cefoxitin, benzylpenicillin but varied in their resistance to fusidic acid (n = 71), erythromycin (n = 16), inducible clindamycin resistance (n = 15), constitutive clindamycin resistance (n = 1), ciprofloxacin (n = 11), trimethoprim (n = 2), gentamicin (n = 2), kanamycin (n = 1), chloramphenicol (n = 1), tetracycline (n = 1) and low-level resistance Mupirocin (n = 1). All isolates were susceptible to vancomycin (MIC ≤ 2 µg/mL), teicoplanin (MIC ≤ 2 µg/mL), linezolid (MIC ≤ 4 µg/mL), and rifampicin. Twenty-one (24.1%) of the CC45 MRSA isolates were resistant to three or more classes of antibiotics and were described as multiply resistant.

The penicillin-resistant CC45 isolates carried the bla operon (*blaZ*, *blaI*, *blaR*) (n = 80). Fusidic acid-resistant isolates were positive for *fusC* (n = 71), and those resistant to erythromycin and clindamycin were positive for *erm(C)* (n = 16). Two trimethoprim-resistant isolates were positive for *dfrS1*, and the tetracycline-resistant isolate was positive for *tet(K)* (n = 1). The chloramphenicol-resistant isolate was positive for *fexA*. Two isolates carried the *lnu(A)*, which encodes lincomycin resistance.

### 2.3. Genomic Analysis of CC45-MRSA Isolates

The isolates were assigned to seven genotypes, comprising CC45-MRSA [IV + fus] (n = 36), CC45-MRSA [VI + fus] (n = 30), and CC45-MRSA-IV,Berlin EMRSA (n = 12) as the common genotypes. Genotypes isolated less frequently were CC45/agrIV-MRSA-IV,WA MRSA-23 (n = 4), CC45-MRSA-V [tst1^+^], WA MRSA-4 (n = 1), CC45-MRSA-IV [tst1^+^] (n = 3), and CC45-MRSA-V (n = 1).

**Figure 1:**
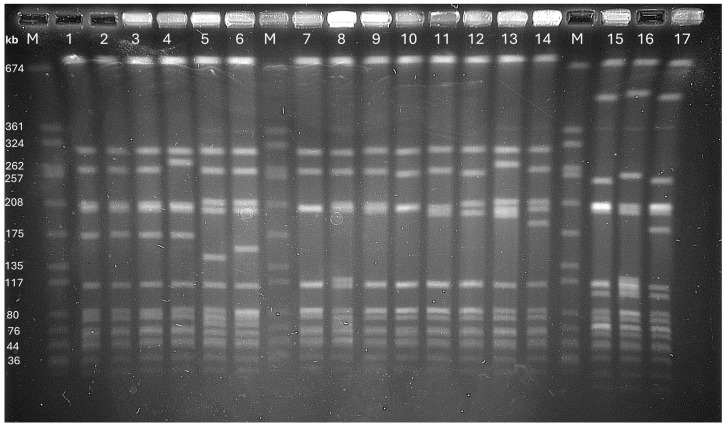
Pulsed-field gel electrophoresis (PFGE) patterns of *SmaI*-digested genomic DNA from CC45-MRSA isolates. M = Molecular size marker (NCTC 8325); Lane 1 + 2 + 3 = PFGE type A; lane 4 = PFGE subtype A9; lane 5 = PFGE subtype A4; lane 6 = PFGE subtype A8; lane 7 = PFGE subtype A2; lane 8 = PFGE subtype A6; lane 9 = PFGE subtype A3; lane 10 = PFGE subtype A2; lane 11 = PFGE subtype A7; lane 12 = PFGE subtype A1; lane 13 = PFGE subtype A10, lane 14 = PFGE subtype A5, lane 15 = PFGE type B, lane 16 = PFGE subtype B1, lane 17 = PFGE subtype B2.

*Spa* typing assigned the isolates to 19 *spa* types. Two *spa* types, t362 (n = 35) and t132 (n = 27), were the most prevalent. *Spa* type, t004, was detected in three isolates. Six *spa* types, t015, t026, t511, t330, t1081, and t1575, were each detected in two isolates. The remaining ten *spa* types, t371, t4449, t4981, t701, t282, t2397, t065, t040, t050 and t0501 were detected in single isolates. MLST classified the isolates into six sequence types: ST7119 (n = 62), ST508 (n = 11), ST45 (n = 4), ST46 (n = 8), and two new sequence types, ST9548 (n = 1) and ST10699 (n = 1). The isolates were further typed by pulsed-field gel electrophoresis (PFGE), which revealed two major PFGE patterns, designated types A and B, along with their respective subtypes (Figure 1; Table 1). A total of 83 isolates belonged to PFGE type A and its 10 subtypes (A1–A10), while four isolates belonged to PFGE type B and its subtypes (B1, B2).

All isolates were positive for the following virulence genes: accessory gene regulator type 1 (*agrI*), capsular polysaccharide type 8 (*cap8*), intercellular adhesion protein A (*icaA*), intercellular adhesion protein C (*icaC*), intercellular adhesion protein D (*icaD*), haemolysin (*hla*, *hlb*, *hld*), leukocidins *(lukS*, *lukF*, *lukX*, *lukY*), and enterotoxin gene cluster, egc, consisting of *seg*, *sei*, *selm*, *seln*, *selu*. They were also positive for staphylokinase (*sak*), chemotaxis-inhibiting protein (*chp*), and staphylococcal complement inhibitor (*scn*), except for six isolates, which were negative.

### 2.4. Distribution of CC45-MRSA Isolates in 2016–2022

The distribution of CC45-MRSA isolates by year of isolation and their genotypic characteristics is presented in Table 2. The CC45-MRSA [IV + fus] and CC45-MRSA [VI + fus] genotypes were isolated throughout the study period (2016–2022). CC45-MRSA-IV,Berlin EMRSA isolates were obtained in five years (2016, 2018, 2019, 2020, and 2021). Isolates belonging to CC45-/agrIV-MRSA-V,WA-MRSA-23, CC45-MRSA-IV [tst1^+^] and CC45-MRSA-V [tst1^+^],WA-MRSA-4, were detected sporadically. Statistical analysis revealed no significant increase in CC45 isolates over the study period (*p*-value > 0.05).

### 2.5. Characteristics of the CC45-MRSA Isolates

#### 2.5.1. CC45-MRSA [IV + Fus] (n = 36)

This genotype consisted of 36 isolates assigned to seven *spa* types: t132 (n = 27), t362 (n = 3), t330 (n = 2), and one of t026, t1575, t371, and t4449. The isolates were obtained from nasal swabs (n = 16), blood (n = 4), wound swabs (n = 4), groin swabs (n = 3), high vaginal swabs (n = 3), and one each of endotracheal swab, throat swab, sputum, and three unspecified sources in eight hospitals. The 36 isolates belonged to two MLST sequence types, ST7119 (n = 33) and ST46 (n = 3), and a single PFGE pattern (Type A and A2).

All isolates resistant to benzylpenicillin and cefoxitin carried *blaZ* and *mecA*. The fusidic acid-resistant isolates were positive for *fusC*. In addition, six isolates were resistant to erythromycin and clindamycin, mediated by *erm(C)*. One isolate was resistant to tetracycline due to *tet(K).* Two isolates were positive for *lnu(A)* (Lincosaminide nucleotidyltransferase) that mediates resistance to lincomycin.

#### 2.5.2. CC45-MRSA [VI + fus] (n = 30)

The CC45-MRSA [VI + fus] genotype comprised 30 isolates obtained from nasal swabs (n = 12), skin and soft tissues (n = 7), throat swabs (n = 2), groin swabs (n = 2), ear swab (n = 3), sputum (n = 1), tracheal aspirate (n = 1), high vaginal swab (n = 1), and an unspecified specimen, collected in 2016, 2017, 2018, 2019, 2020, 2021, and 2022 from eight hospitals. The 30 isolates were resistant to cefoxitin, benzylpenicillin, and fusidic acid, mediated by *mecA*, *blaZ*, and *fusC*, respectively. Six isolates were resistant to erythromycin, with inducible clindamycin resistance mediated by *erm(C).* One isolate was resistant to chloramphenicol, and another to ciprofloxacin.

*Spa* typing revealed that 28 isolates belonged to t362, while the remaining two isolates belonged to t701 and t282. The 30 isolates belonged to PFGE type A and its subtypes (Table 1), and two sequence types, ST7119 (n = 29) and ST46 (n = 1).

#### 2.5.3. CC45-MRSA-IV, Berlin EMRSA (n = 12)

The 12 isolates identified as CC45-MRSA-IV, Berlin EMRSA, were cultured from four nasal swabs, three endotracheal aspirates, two blood samples, and one each from groin, aspiration fluid, and an unspecified source. All 12 isolates were resistant to cefoxitin and benzylpenicillin mediated by *mecA* and *blaZ*, respectively. Two isolates were resistant to erythromycin and clindamycin, mediated by *erm(C).* Three isolates were resistant to fusidic acid, mediated by *fusC*, and two isolates were resistant to ciprofloxacin. The isolates belonged to seven *spa* types, consisting of t362 (n = 3), t004 (n = 3), t511 (n = 2) and one of t040, t050, t0510 and t1575. They belonged to four sequence types: ST508 (n = 7), ST46 (n = 3), including two novel STs (ST9548 and ST10699), and seven PFGE subtype A (Table 1). Seven isolates were positive *for sak*, *chp*, *and scn*, and five isolates were negative for all three genes. The 12 isolates were positive for *egc*, while five isolates were positive for *seb*.

#### 2.5.4. CC45/agrIV-MRSA-IV,WA MRSA-23 (n = 4)

Four isolates cultured from three nasal swabs and one skin swab collected in 2017, 2019, and 2021 at three hospitals were identified as CC45/agrIV-MRSA-IV WA MRSA-23. The four isolates were resistant to cefoxitin and benzylpenicillin, mediated by *mecA* and *blaZ*, respectively. Three of them were susceptible to the non-beta-lactam antibiotics tested. One isolate was resistant to ciprofloxacin. The isolates belonged to a single sequence type, ST45, one PFGE type, subtype B (Table 1), but to three *spa* types: t1081 (n = 2), t4981 (n = 1), and t026 (n = 1). All four isolates were positive for *sasG*, which encodes the *Staphylococcus aureus* surface protein G.

#### 2.5.5. CC45-MRSA-IV [tst1^+^] (n = 3)

Three isolates cultured from nasal swabs, tissue, and an unspecified sample at two hospitals in 2018 (n = 2) and 2019 (n = 1) were identified as CC45-MRSA-IV [tst1^+^]. Two of the isolates were resistant to erythromycin and clindamycin, mediated by *erm(C).* They belonged to two *spa* types: t015 (n = 2) and t2397. All three isolates belonged to the same sequence type, ST508 and PFGE subtype A2. The isolates were positive for *agrI*, *cap8*, *sak*, *chp*, *scn*, *egc*, and *tst1*.

#### 2.5.6. CC45-MRSA-V-[tst1^+^],WA MRSA-4 (n = 1)

This genotype was identified in a single isolate obtained from a 2019 semen sample. It was resistant to trimethoprim and fusidic acid mediated by *dfrS1* and *fusC*, respectively. It was positive for *agrI*, *cap8*, *sak*, *chp*, *scn*, *egc*, and *tst1*. It belonged to *spa* type t362, PFGE subtype A2, and ST508.

#### 2.5.7. CC45-MRSA-V (n = 1)

This genotype was identified in a single isolate cultured from a nasal swab in 2021. It was resistant to fusidic acid mediated by *fusC*. It belonged to *spa* type, t065, PFGE subtype A4, and ST46. It was positive for *agrI*, *cap8*, *icaA*, *icaC*, *icaD*, and *egc*, but negative for s*ak*, *chp*, and *scn*.

## 3. Discussion

This study investigated antibiotic resistance and genotypic diversity among CC45-MRSA isolates from human clinical samples in Kuwait. MRSA isolates belonging to Clonal Complex 45 (CC45) have been widely reported in North America [28], Australia [29,30], and Germany [31], but have occurred sporadically in Middle Eastern and Gulf Cooperation Council (GCC) countries, including Kuwait [27,32]. The low prevalence of CC45-MRSA in this study (87 isolates, 0.65%) aligns with reports from the UAE [33,34], Lebanon [35], Saudi Arabia [36,37], and Tunisia [38], confirming its low prevalence in the region, in contrast to the high prevalence of CC45-MRSA among patients in long-term care facilities in Singapore [39] and among healthy community members in Northern Vietnam [40].

Antibiotic susceptibility testing showed that most CC45-MRSA isolates were susceptible to most antibiotics tested. This aligns with studies from Poland [41], the UAE [34], China [42], and Uganda [43]. However, 24.1% of isolates were multidrug resistant (MDR), showing resistance to multiple antibiotics, including penicillin, fusidic acid, erythromycin, clindamycin, ciprofloxacin, trimethoprim, gentamicin, kanamycin, chloramphenicol, and tetracycline. These MDR CC45 isolates were not associated with a specific genotype (*p*-value > 0.05).

Previous studies have reported that CC45-MRSA isolates belong to diverse genotypes and carry different SCC*mec* types, including USA600 (ST45-MRSA-II), the Berlin epidemic clone (ST45-MRSA-IV) [4,5,11], and ST45-MRSA-V [44]. Similarly, the CC45-MRSA isolates in this study exhibited diversity in SCC*mec* types (IV, V, and VI), *spa* types, and sequence types (Table 1). Although the USA600 (ST45-MRSA-II) was not detected in this study, seven CC45-MRSA variants were detected, including CC45-MRSA [VI + fus], CC45-MRSA [IV + fus], CC45-MRSA-IV,Berlin EMRSA, CC45-/agrIV-MRSA-IV,WA MRSA-23, CC45-MRSA-IV [tst1^+^],WA MRSA-4, CC45-MRSA-V [tst1^+^], and CC45-MRSA-V. CC45-MRSA-[IV + fus] (41.3%), CC45-MRSA-[VI + fus] (34.4%), and CC45-MRSA-IV, Berlin EMRSA (13.7%) were the dominant genotypes. The other genotypes were detected sporadically. The diverse composition of the CC45-MRSA population in this study suggests multiple routes of introduction into the country.

Most previously reported CC45-MRSA isolates have belonged to ST45 [4,5,7,45,46], whereas most (71.2%) of the CC45-MRSA isolates in the present study belonged to ST7119, followed by ST508 (12.6%), ST46 (9.2%), ST45 (4.6%), and the novel STs: ST9548 (1.1%) and ST10699 (1.1%) (Table 1). Other studies have reported ST508 and ST929 as major components of CC45 isolates in Taiwan [19]. Nowrouzian et al. [46] reported that the CC45 population in Sweden consisted of ST45, ST46, and ST455. Additionally, da Silva et al. [47] reported that CC45-MRSA isolates colonizing healthcare workers in a Brazilian hospital comprised ST45 and ST1914. However, this is the first report of ST7119, ST9548, and ST10699 among *S. aureus* isolates from Kuwait hospitals, thereby further increasing the genetic diversity of CC45-MRSA isolates.

The ST7119 isolates belong to the two dominant genotypes, CC45-MRSA [IV + fus] and CC45-MRSA [VI + fus], with the majority belonging to *spa* types t362 and t132 (Table 1). Prior to this report, ST7119 was reported from a single isolate in the Netherlands [48]. Because the *spa* and SCC*mec* types of the ST7119 isolate from the Netherlands were not provided, it is difficult to establish its epidemiologic relationship with those in this study. Additionally, neither CC45-MRSA [VI + fus] nor ST7119 has been previously reported in the United Arab Emirates, Saudi Arabia, or other Gulf Cooperative Council (GCC) countries, suggesting that these isolates may have emerged locally in Kuwait.

Furthermore, CC45-MRSA [IV + fus], the dominant genotype in this study, has been reported sporadically in the United Arab Emirates [33,34] and Saudi Arabia [37], underscoring its rarity in the GCC region. The apparent higher numbers of CC45-MRSA [IV + fus] isolates reported in this study are likely due to the isolates being collected over seven years. The lack of significant year-to-year increases in their numbers suggests that they are unable to spread readily. It is notable that CC45-MRSA [IV + fus] isolates belonging to t132 were obtained from household cattle and buffalo in Egypt [25], suggesting that this may also be a Livestock-Associated MRSA genotype. There are currently no data on the colonization of livestock in Kuwait by CC45 MRSA isolates. This warrants investigations to determine if there is an animal reservoir for these strains in Kuwait.

Significantly, the two dominant genotypes, CC45-MRSA [IV + fus] and CC45-MRSA [VI + fus], are resistant to fusidic acid mediated by *fusC* and are responsible for the high prevalence of fusidic acid resistance observed in this study. *fusC* is part of the composite genetic elements, SCC*mec* IV + fus and SCC*mec* VI + fus. The carriage of the composite genetic element SCC*mec* VI + fus in these CC45-MRSA isolates appears to follow patterns observed in CC30-MRSA-[VI + fus] [34,37,49], CC22-MRSA [VI + fus] [34,50], CC5-MRSA [VI + fus] [51,52], CC97-MRSA [VI + fus] [49], and CC8-MRSA [VI + fus] [34,53]. These composite genetic elements carrying the fusidic acid resistance determinant, *fusC*, may confer a survival advantage in the presence of fusidic acid, which may help explain the troublingly high prevalence of fusidic acid resistance among MRSA isolates in the GCC region [34,37,51,53,54]. High prevalence of fusidic acid resistance is a growing problem among MRSA isolates in Kuwait [27,51,53,54] and other GCC countries [33,34,37]. This warrants a review of guidelines governing access to and use of fusidic acid in the region.

The CC45-MRSA-IV,Berlin EMRSA was the third most common genotype in this study. The 12 isolates belonged to four STs, including ST508 and ST46, previously reported [19,46,47], and two novel STs, ST9548 and ST10699, reported in this study. The detection of two novel STs in this study adds to the growing diversification of the Berlin EMRSA lineage. These isolates also belonged to diverse *spa* types, including t004 and t040, which were associated with the original Berlin EMRSA strains in Germany [11], as well as t362, t050, t1575, t511, and t0510, which were described in other studies [45,55,56,57]. The isolates also belonged to seven PFGE subtypes, underscoring the diversity of Berlin EMRSA in this study. The Berlin EMRSA lineage first appeared as a single arginine catabolic mobile element (ACME)-positive ST508-MRSA-IV-t050 isolate in Kuwait during 2001–2005 [27] but was not reported again until 2016. Notably, all isolates examined in this study were negative for ACME, suggesting that the current isolates are distinct from those reported in 2001–2005 [27]. The detection of the Berlin EMRSA in 14 isolates over 17 years (2005–2022) indicates a low transmission of this strain in Kuwait hospitals.

The CC45/agrIV-MRSA-IV,WA MRSA-23 genotype was detected in four isolates in this study. CC45/agrIV-MRSA-IV,WA MRSA-23 isolates have also been reported in small numbers in Saudi Arabia [37,58], underscoring the low prevalence of these genotypes in the GCC countries. Notably, only the CC45/agrIV-MRSA-IV,WA MRSA-23 genotype belonged to ST45, representing the parental CC45 lineage in this study, whereas the other genotypes were single-locus variants (SLVs) or double-locus variants (DLVs) (ST7119 and ST9548) of ST45 (Table 1). The CC45/agrIV-MRSA-IV,WA MRSA-23 was the only genotype that was positive for *sasG*, as reported for Australian isolates [4]. The isolates in this study were associated with *spa* types t1081, t026, and t4981. Similarly, t1081 was associated with CC45/agrIV-MRSA-IV in Australia [4,22,59], Hong Kong [60,61], and Taiwan [19], and was common among human and animal isolates in Uganda [43]. In addition, the current isolates, as well as those obtained in Australia, Hong Kong, and Taiwan, carry the gene for type 8 capsular polysaccharide, *cap8*, and therefore appear to be related to the Australian rather than the African group of CC45, which carries *cap5* [5]. The uniqueness of the CC45/agrIV-MRSA-IV,WA MRSA-23 genotype was confirmed by PFGE results, which clustered the isolates into a single PFGE pattern (type B) (Figure 1).

Four isolates comprising three CC45-MRSA-IV [tst1^+^], and one CC45-MRSA-V [tst1^+^], WA-MRSA-4 were positive for *tst1*, the gene encoding toxic shock syndrome toxin. The four isolates belonged to ST508, shared the same PFGE pattern, but differed in *spa* types (Table 1). Similarly, a few CC45-MRSA-IV/V isolates from Australia and Europe have been shown to carry *tst1* [4,5]. The other CC45-MRSA-V isolate detected in this study was tst1-negative and belonged to ST46 and t065. This isolate may be related to the Australian genotype, WA-MRSA-84 [4,59].

Analysis of virulence factors showed that CC45 isolates carried fewer enterotoxins, yet all contained the enterotoxin gene cluster (*egc*), as reported elsewhere [4,62,63].

Limitations of this study include a lack of demographic data on patients’ age and gender, which would help determine whether CC45-MRSA isolates disproportionately affect specific patient groups. It also lacks clinical information regarding active infection versus colonization, treatments, and outcomes. Nevertheless, isolation of the organisms from various clinical samples, including blood cultures, suggests that the CC45-MRSA isolates can colonize and cause invasive infections, as reported in other studies [5,6,7].

## 4. Materials and Methods

### 4.1. Collection of MRSA Isolates

MRSA isolates were collected from 13,276 unique patients from 2016 to 2022 in 13 hospitals in Kuwait as part of routine diagnostic microbiology services. The isolates were obtained from various clinical samples. Initial identification of MRSA isolates was performed in diagnostic microbiology laboratories using the VITEK MS system (bioMérieux, Marcy l’Etoile, France). The isolates were then sent to the Gram-Positive Bacteria Research laboratory in the Department of Microbiology, College of Medicine, Kuwait University, where they were retested for purity and preserved in 40% glycerol (*v*/*v*) in brain heart infusion broth at −80 °C for further analysis.

### 4.2. Antimicrobial Susceptibility Testing

The test was performed by the disc diffusion method using the following antibiotics discs (Oxoid, Basingstoke, UK): benzylpenicillin (2U), cefoxitin (30 μg), kanamycin (30 μg), mupirocin (200 μg), gentamicin (10 μg), erythromycin (15 μg), clindamycin (2 μg), chloramphenicol (30 μg), tetracycline (10 μg), trimethoprim (2.5 μg), fusidic acid (10 μg), rifampicin (5 μg), ciprofloxacin (5 μg), teicoplanin (30 μg) and linezolid (30 μg). Vancomycin, cefoxitin, teicoplanin, linezolid, and mupirocin were tested for minimum inhibitory concentration (MIC) using E-test strips (bioMérieux, Marcy-l’Étoile, France). The isolates were categorized as susceptible, intermediate, or resistant according to the Clinical Laboratory Standards Institute [64].

### 4.3. DNA Microarray Analysis

The DNA microarray was performed using the INTER-ARRAY Genotyping Kit *S. aureus* (Inter-Array GmbH, Bad Langensalza, Germany) according to the manufacturer’s protocol and as previously described [11]. The microarray analysis was used to assign clonal complexes (CCs) and to determine the carriage of genes encoding antibiotic resistance and virulence factors.

### 4.4. Staphylococcal Protein A (Spa) Typing

*Spa* typing was performed according to the previously published protocol and primers [65]. Amplification of the *Spa* gene was performed using synthetic primers [66] in a total volume of 25 μL. The PCR protocol consisted of an initial denaturation at 94 °C for 4 min, followed by 25 cycles of denaturation at 94 °C for 1 min, annealing at 56 °C for 1 min, extension at 72 °C for 3 min, and a final extension at 72 °C for 5 min. Five μL of the PCR product was analyzed by 1.5% agarose gel electrophoresis to confirm amplification. The *spa* gene sequence was analyzed using Ridom Staph Type software v. 2.2.1 (Ridom GmbH, Würzburg, Germany).

### 4.5. Multi-Locus Sequence Typing (MLST)

MLST was performed as described by Enright et al. [66] on representative isolates using M13-tailed primers (Applied Biosystem, Carlsbad, CA, USA). Isolates for MLST were selected based on their *spa* types. MLST involves obtaining the sequences of internal fragments of seven housekeeping genes for each *S. aureus* strain. The following housekeeping genes were used: Carbamate kinase (*arcC*), Shikimate dehydrogenase (*aroE*), Glycerol kinase (*glpF*), Guanylate kinase (*gmk*), Phosphate acetyltransferase (*pta*), Triosephosphate isomerase (*tpi*), Acetyl coenzyme A acetyltransferase (*yqiL*). The Sequence types (STs) were determined by obtaining the allele number for each housekeeping gene [67].

### 4.6. Pulsed-Field Gel Electrophoresis (PFGE)

PFGE was performed using Contour-clamped Homogeneous Electric Field (CHEF) electrophoresis [68]. A loopful of overnight bacterial culture on blood agar plates was washed three times with 1.0 mL of 50 mM EDTA (pH 8.0) and centrifuged at 13,000 rpm for 3 min. The pellet was suspended in 0.5 mL of EC buffer (6 mM Tris–HCl, 1 M NaCl, 100 mM EDTA, 0.2% Sodium deoxycholate) to prepare a bacterial suspension equivalent to 12 × 10^8^ CFU/mL. The casting blocks were prepared by mixing 100 μL of the bacterial suspension with 50 μL of lysostaphin (400 mg/mL) and 150 μL of 1.5% agarose (chromosomal grade, Bio-Rad Laboratories, Richmond, CA, USA), then transferring the mixture to PFGE block molds (Bio-Rad, Hercules, CA, USA). The formed blocks were pushed into a microfuge tube containing 1.0 mL of cell lysing solution and incubated for 3–4 h in a water bath at 37 °C. After incubation, the cell lysing buffer was replaced with proteinase K buffer (20 mg/mL) and the mixture was incubated overnight in a water bath at 55 °C. The blocks were washed once with 1.0 mL TE buffer (1 mM Tris, 0.2 mM EDTA, pH 8.0) in a water bath at 64 °C for 1 h, followed by three washes with sterile distilled water for 30 min, each at 37 °C water bath with occasional shaking. The blocks were digested with 40U *Sma*I (New England Biolabs, Ipswich, Rowley, MA, USA) for 3 h at 25 °C in a water bath. The digested blocks were loaded into a 1.2% (*w*/*v*) pulsed-field-grade agarose gel (Pulsed-field certified agarose, BioRad, Hercules, CA, USA) and run for 22 h with an initial pulse of 5 s and a final pulse of 40 s in the CHEF-DR III system (BioRad, Hercules, CA, USA). *Sma*I-digested *S. aureus* strain NCTC 8325 was used as a molecular size marker. The gel was stained with 0.5 µg/mL ethidium bromide and photographed under UV illumination. Strain-relatedness was determined according to the criteria defined by Tenover et al. [69].

### 4.7. Statistical Analysis

Statistical analysis was performed using SPSS (Statistical Package for the Social Sciences), version 25 (SPSS Inc., Chicago, IL, USA), to determine whether there was a significant trend (increase or decrease) in CC45-MRSA isolates over time, using linear regression, and to establish a correlation between genotypes and MDR isolates using Pearson chi-square. A *p*-value of <0.05 was considered statistically significant.

## 5. Conclusions

In conclusion, our results confirm a low prevalence of CC45-MRSA isolates in Kuwaiti hospitals. However, these isolates comprise diverse genotypes, dominated by ST7119 (DLV of ST45), which carries either SCC*mec* IV + fus or SCC*mec* VI + fus, both of which are rare in other countries. Other genotypes isolated less frequently included CC45-MRSA-IV,Berlin EMRSA, CC45-/agrIV-MRSA-IV,WA MRSA-23, CC45-MRSA-IV [tst1^+^], WA MRSA-4, CC45-MRSA-V [tst1^+^], and CC45-MRSA-V. The dominance of the ST7119-CC45-MRSA-[IV + fus] and ST7119-CC45-MRSA [VI + fus] genotypes correlated with a high prevalence of fusidic acid resistance mediated by *fusC.* Fusidic acid resistance is a growing problem among MRSA populations in Kuwait [50,51,54]. In addition, 24.1% of the CC45-MRSA isolates in this study displayed multidrug resistance. The emergence of multidrug resistance among CC45-MRSA isolates in Kuwaiti hospitals is concerning because it limits treatment options, prolongs hospital stays, and increases complication rates in MRSA infections. The diverse genetic backgrounds observed in this study suggest multiple transmission routes for CC45 lineages in Kuwait and highlight the importance of developing regional surveillance across the Gulf Cooperation Council (GCC) countries to formulate effective infection control strategies and antimicrobial stewardship in healthcare settings.

## Figures and Tables

**Table 1:** Genotypic characteristics of CC45-MRSA isolates.

No.	Genotypes (N)	*Spa* Types	PFGE Types	MLST	*arcC*	*aroE*	*glpF*	*gmK*	*pta*	*tpi*	*yqil*	N
1	CC45-MRSA [IV + fus] (36)	t132	A	ST7119	10	14	8	6	14	3	615	27
		t026	A	ST7119	10	14	8	6	14	3	615	1
		t1575	A	ST7119	10	14	8	6	14	3	615	1
		t4449	A	ST7119	10	14	8	6	14	3	615	1
		t362	A	ST7119	10	14	8	6	14	3	615	3
		t371	A2	ST46	10	14	8	6	14	3	2	1
		t330	A2	ST46	10	14	8	6	14	3	2	2
2	CC45-MRSA [VI + fus] (30)	t362	A1, A2, A3, A7	ST7119	10	14	8	6	14	3	615	28
		t701	A1	ST7119	10	14	8	6	14	3	615	1
		t282	A1	ST46	10	14	8	6	14	3	2	1
3	CC45-MRSA-IV, Berlin EMRSA (12)	t362	A1	ST46	10	14	8	6	14	3	2	1
		t362	A2	ST508	10	40	8	6	10	3	2	2
		t040	A2	ST10699	10	14	8	6	1233	3	2	1
		t004	A6	ST46	10	14	8	6	14	3	2	1
		t004	A6	ST46	10	14	8	6	14	3	2	1
		t050	A5	ST508	10	40	8	6	10	3	2	1
		t1575	A5	ST508	10	40	8	6	10	3	2	1
		t511	A4	ST508	10	40	8	6	10	3	2	2
		t0510	A8	ST9548	108	40	8	6	14	3	2	1
		t004	A10	ST508	10	40	8	6	10	3	2	1
4	CC45/agrIV-MRSA-IV, WA-MRSA-23 (4)	t1081	B	ST45	10	14	8	6	10	3	2	2
		t026	B1	ST45	10	14	8	6	10	3	2	1
		t4981	B2	ST45	10	14	8	6	10	3	2	1
5	CC45-MRSA-V [tst1^+^], WA MRSA-4 (1)	t362	A2	ST508	10	40	8	6	10	3	2	1
6	CC45-MRSA-IV[tst1^+^] (3)	t2397	A2	ST508	10	40	8	6	10	3	2	1
		t015	A2	ST508	10	40	8	6	10	3	2	2
7	CC45-MRSA-V (1)	t065	A4	ST46	10	14	8	6	14	3	2	1

**Abbreviations**: *arc* (Carbamate kinase); *aroE* (Shikimate dehydrogenase); *glpF* (Glycerol kinase); *gmk* (Guanylate kinase); *pta* (Phosphate acetyltransferase); *tpi* (Triosephosphate isomerase); *yqi*L (Acetyl-Coenzyme A acetyltransferase).

**Table 2:** Distribution of CC45 MRSA isolates per year.

Genotypes	2016	2017	2018	2019	2020	2021	2022	Total
CC45-MRSA [IV + fus]	5	3	2	1	6	8	11	36
CC45-MRSA [VI + fus]	2	7	11	3	3	1	3	30
CC45-MRSA-IV, Berlin EMRSA	1	0	1	1	5	4	0	12
CC45-MRSA-V [tst1^+^], WA-MRSA 4	0	0	0	1	0	0	0	1
CC45-/agrIV-MRSA-V, WA-MRSA 23	0	2	0	1	0	1	0	4
CC45-MRSA-IV [tst1^+^]	0	0	2	1	0	0	0	3
CC45-MRSA-V	0	0	0	0	0	1	0	1
Total	8	12	16	8	14	15	14	87

## Data Availability

All data generated for this study are included in this article.
